# Neurotransmitter Dysfunction in Irritable Bowel Syndrome: Emerging Approaches for Management

**DOI:** 10.3390/jcm10153429

**Published:** 2021-07-31

**Authors:** Mónica Gros, Belén Gros, José Emilio Mesonero, Eva Latorre

**Affiliations:** 1 Centro de Salud Univérsitas, Hospital Clínico Universitario Lozano Blesa, 50009 Zaragoza, Spain; mgrosalc@gmail.com; 2 Instituto de Investigación Sanitaria de Aragón (IIS Aragón), 50009 Zaragoza, Spain; grosbel@gmail.com (B.G.); mesonero@unizar.es (J.E.M.); 3 Servicio de Urgencias, Hospital Universitario Miguel Servet, 50009 Zaragoza, Spain; 4 Departamento de Farmacología, Fisiología y Medicina Legal y Forense, Facultad de Veterinaria, Universidad de Zaragoza, 50009 Zaragoza, Spain; 5 Instituto Agroalimentario de Aragón—IA2—(Universidad de Zaragoza—CITA), 50013 Zaragoza, Spain; 6 Departamento de Bioquímica y Biología Molecular y Celular, Facultad de Ciencias, Universidad de Zaragoza, 50009 Zaragoza, Spain

**Keywords:** IBS, microbiota, visceral hypersensitivity, colorectal motility

## Abstract

Irritable bowel syndrome (IBS) is a functional gastrointestinal disorder whose aetiology is still unknown. Most hypotheses point out the gut-brain axis as a key factor for IBS. The axis is composed of different anatomic and functional structures intercommunicated through neurotransmitters. However, the implications of key neurotransmitters such as norepinephrine, serotonin, glutamate, GABA or acetylcholine in IBS are poorly studied. The aim of this review is to evaluate the current evidence about neurotransmitter dysfunction in IBS and explore the potential therapeutic approaches. IBS patients with altered colorectal motility show augmented norepinephrine and acetylcholine levels in plasma and an increased sensitivity of central serotonin receptors. A decrease of colonic mucosal serotonin transporter and a downregulation of α2 adrenoceptors are also correlated with visceral hypersensitivity and an increase of 5-hydroxyindole acetic acid levels, enhanced expression of high affinity choline transporter and lower levels of GABA. Given these neurotransmitter dysfunctions, novel pharmacological approaches such as 5-HT_3_ receptor antagonists and 5-HT_4_ receptor agonists are being explored for IBS management, for their antiemetic and prokinetic effects. GABA-analogous medications are being considered to reduce visceral pain. Moreover, agonists and antagonists of muscarinic receptors are under clinical trials. Targeting neurotransmitter dysfunction could provide promising new approaches for IBS management.

## 1. Introduction

Irritable bowel syndrome (IBS) is defined as a functional gastrointestinal disorder, whose main symptoms are recurrent abdominal pain, changes in the frequency or characteristics of stool and abdominal distension. As a functional gastrointestinal disorder, IBS does not have a morphologic, metabolic, or neurologic aetiology. It is diagnosed using Rome IV clinical parameters. IBS can be classified in 4 different subtypes according to patient’s bowel habit: IBS with predominant constipation (IBS-C), IBS with predominant diarrhoea (IBS-D) and mixed-IBS which alternates between diarrhoea and constipation (IBS-M). Another type of IBS is called unclassified (IBS-U) [1], where individuals who do not fall into the other intestinal pattern categories are included.

IBS is considered the most prevalent gastrointestinal disorder; its prevalence is estimated to be around 10% to 15% of the population in Europe and North America. Despite its high prevalence, the physiopathology of IBS is still unknown. There are many hypotheses about IBS aetiology: psychosocial disorders, microbiotic alterations, hypersensitivity to some food, intestinal motility disorders, changes in visceral pain perception, or neurotransmitter alterations, creating a complex disorder of the gut-brain axis [2]. This axis is composed of intestinal microbiota, the intestinal epithelial barrier, neurotransmitters, the central nervous system (CNS), enteric nervous system (ENS), autonomic nervous system, and the hypothalamic-pituitary-adrenal axis. Together, all these components communicate bidirectionally (mainly through neurotransmitters), so intestinal signals can influence brain functions and vice versa. In fact, IBS patients show differences in brain activation areas in response to rectal distension and pain compared with healthy controls; suggesting that IBS patients lack central activation of descending inhibitory pathways [3]. Recent studies have reported alterations in brain networks and networks of interacting systems in the gut in IBS patients, evidencing a potential role of neurotransmitters on IBS pathophysiology [4]. On the other hand, psychosocial factors such as stress, anxiety, or depression, where neurotransmitters can play a key role, are considered risk factors for IBS and may even contribute to an exacerbation of IBS symptoms [5].

In recent years, many studies have focused on the association between IBS and changes in gut microbiota [6]. Gut microbiota can modulate host production of different neurotransmitters, as well as produce some neurotransmitters themselves [7]. Gut microbiota could play a role in the aetiology of IBS as they influence intestinal motility, gastrointestinal physiology, neurotransmitter levels, and behaviour. Actually, germ-free rats display a delay in intestinal peristalsis and that can be reverted by colonization with *Lactobacillus acidophilus* or *Bifidobacterium bifidum* [8]. As demonstrated in several studies, IBS patients show perturbed microbiota composition, although there is no common microbiotic signature among IBS patients [9]. An increase of *Firmicutes*, especially *Clostridium* and *Ruminococcaceae* with a decrease of *Bacteroidetes*, particularly *Bifidobacteria* can be obtained in several mucosal and faecal samples from IBS patients [10]. Moreover, preliminary data suggest correlations of regional brain structural differences with gut microbial taxa [4].

**Figure 1:**
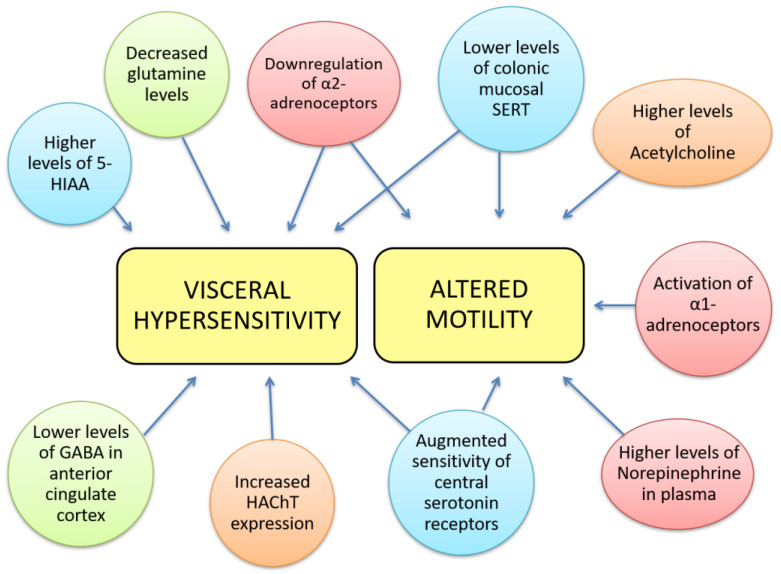
Neurotransmitter dysfunctions are related to some gastrointestinal IBS symptoms. Visceral hypersensitivity has been correlated to decreased glutamine levels, lower levels of GABA in the anterior cingulate cortex, higher levels of 5-hydroxy-indol acetic acid, increased expression of high affinity choline transporter, downregulation of α-2 adrenoceptors, augmented sensitivity of central serotonin receptors and lower levels of mucosal SERT. The latter 3 alterations can also be found in altered colorectal motility together with higher levels of NE in plasma, activation of α-1 adrenoceptors and higher levels of ACh. We notate neurotransmitter’s families with colours: red- norepinephrine; blue- 5-HT; green- GABA; orange-acetylcholinergic.

The pathophysiology of IBS is incompletely understood, but it is well established that alterations in the gut-brain axis, altered CNS processing, motility disturbances and visceral hypersensitivity contribute to IBS aetiology. Other, less relevant or less studied mechanisms involved in IBS include genetic associations, alterations in gastrointestinal microbiota, cultural factors, and disturbances in mucosal and immune function [11]. Alterations in the gut-brain axis and differences in brain function are major contributing factors to IBS aetiology; however, the implications of key neurotransmitters such as norepinephrine (NE), serotonin, glutamate, GABA, and acetylcholine (ACh) in IBS are still unknown. The aim of this review is to evaluate the current evidence about neurotransmitter dysfunction in IBS and explore its potential therapeutic treatment. The Rome IV criteria for the diagnosis of IBS consist of abdominal pain associated with an alteration in either stool form or frequency, occurring for at least 6 months. Neurotransmitter dysfunctions could contribute to IBS and some of its most prevalent symptoms used for its diagnosis, grouped into two main aspects, visceral hypersensitivity and altered motility (Figure 1), although they may also be involved in other symptoms such as diet-related digestive disturbances, psychosocial disturbances, anxiety, depression, fatigue, hypertension, dyslipidaemia, etc. Therefore, targeting those dysfunctions may open novel lines for IBS management, taking into account, that these symptoms may also be indirect effects mediated by other biological and psychological factors.

## 2. Norepinephrine

NE, also known as noradrenaline, is a key catecholamine with multiple physiological and homeostatic functions, key in the sympathetic nervous system. It is involved in excitation and the alert state during awake time, and in sensory signal detection. Secondarily, NE plays a role in behaviour, memory, attention, and learning. In fact, NE depletion in rats triggers distractibility and attentional deficits [12]. NE also has a leading role in spatial working, and memory functions, and its level is correlated with cognitive performance.

### 2.1. Norepinephrine in the Central Nervous System

Noradrenergic neurons come from the locus coeruleus, and their axons reach many brain regions. NE improves long-term memory consolidation, influences the processing of sensory stimuli in the amygdala and hippocampus, and also regulates working memory and attention in the prefrontal cortex [13]. There are 3 types of adrenergic receptors, which NE can interact when is released from ascending fibres: the stimulatory α1 and β adrenoceptors, and the inhibitory α2 adrenoceptor. Among those receptors, NE has a higher affinity for α2, which has 3 subtypes: α2A, α2B and α2C. Although α2-adrenoceptors are found postsynaptically, subtypes α2A and C are predominantly presynaptic [14]. There are also 3 subtypes of α1-adrenergic receptors, α1A, α1B and α1D, for which NE has lower affinity. Stimulation of those receptors enhances excitatory processes, especially in the somatosensory cortex. β-receptors are divided into 3 types: β1, localized in the heart, β2 in the lungs and β3 in stomach and adipose tissues. They are also expressed in the CNS; however, NE has low affinity for these types of receptors. Electromagnetic studies in primates have found β2-receptor expression on dendritic spines in the prefrontal cortex and on GABAergic interneurons; on glia, these β-receptors reduce glutamate reuptake and regulate glucose availability [15]. Similarly, other studies have demonstrated that β-receptors could enhance GABAergic processes in the somatosensory cortex [16].

### 2.2. Norepinephrine’s Role in the Gastrointestinal System

In the peripheral nervous system, noradrenergic neurons respond to stress via sympathetic. Higher levels of epinephrine or NE can increase heart rate (via β1-receptors), pulmonary function (via β2-receptors), and blood pressure (via α1- and β-receptors) to increase the amount of oxygenated blood in striated muscle [17]. Via β3-receptors, digestive function is reduced. However, acute stress in mice stimulates colonic contractile activity almost immediately for defecation [18]. Presynaptic inhibition of NE is the main role of an α2A-adrenoceptor. Decreased levels of this receptor and NE transporter (NET) were found in colon of IBS rats, resulting in an increased release of NE [19]. Moreover, α2A and α2C polymorphisms are associated with constipation and high somatic symptoms in patients with lower functional gastrointestinal disorders [20]. This genetic variation in α2-adrenoceptors could influence not only visceral sensation and stool frequency (especially in IBS-C), but also behaviour in IBS patients. Noradrenaline also seems to affect colorectal motility. The intrathecal injection of noradrenaline induces a propulsive motility through activation of α1-adrenoceptors on sacral parasympathetic preganglionic neurons in rats [21]. In contrast, intrathecal injection of prazosin (α1-adrenoceptor antagonist) presents no effects on colorectal motility, confirming that noradrenergic descending pathway from the brain influences gastrointestinal motility by acting on the lumbosacral spinal defecation centre.

Intestinal NE can increase the pathogenicity of some bacteria. Pathological *Escherichia coli* O157:H7 (EHEC’s) growth is enhanced by the presence of dopamine and NE in intestinal lumen. NE also increases motility, the ability to create biofilm, and virulence of EHEC [22]. In turn, gut microbiota can influence NE levels in intestinal lumen, but it is still undetermined whether bacteria can produce NE themselves or only modulate host production [23]. Germ-free mice show lower levels of NE in caecal tissue. Those mice also present behavioural changes that can be reverted by probiotics. These data support the relation between microbiota and neurotransmitters [24] and highlight microbiota’s role in the gut-brain axis. Using microbiota modulation as a source of neurotransmitters to coordinate neurological function could be an interesting approach to study.

Stress could be a risk factor for IBS development. There are 3 main mediators of stress: corticotropin releasing hormone, corticosterone, and NE. Plasma concentrations of corticosterone and NE were significantly higher after 9-day mild stress in rats [25]. Although it is known that IBS patients usually report higher levels of psychological distress, Deechakawan W. et al. found no relation between the improvement of psychological symptoms and norepinephrine levels in urine [26]. Similarly, other studies revealed no differences in blood cathecolamin levels during sleep. However, differences between IBS subtypes were found: women with IBS-C displayed significantly increased NE, epinephrine and cortisol levels throughout the sleep interval, and women with IBS-D presented lower levels of NE and cortisol [27]. Chronic stress in rats increases α_1C_ subunit of Ca_v_1.2 channels in colonic muscularis. These changes are expressed clinically as accelerated colonic transit and increased defecation rate. Actually, NE induces colonic circular smooth muscle hyperreactivity to acetylcholine [18]. In agreement, an inverse relationship between parasympathetic tone and epinephrine plasma levels in IBS patients has been observed [28]. However, NE alterations are not clear, as some studies have shown higher levels of norepinephrine in blood, urine and saliva in IBS patients [29]. Berman et al. [29] demonstrated that IBS patients had higher plasma NE levels than healthy controls before and after ingestion of yohimbine (α2A-adrenoreceptor antagonist) and clonadine (α2A-adrenoreceptor agonist). That augmentation of noradrenergic activity can be explained by a downregulation of presynaptic inhibitory α2A-receptors. Both phenomena (higher plasma NE levels and downregulation of presynaptic inhibitory α2A-receptors) were correlated with anxiety disorders [30].

Enzymes from the noradrenergic system have also been studied in IBS, including tyrosine hydroxylase (TH), whose function is rate-limiting norepinephrine production. TH expression seems to be increased in IBS-D rats, although the augmentation was nonsignificant [31]. Chronic stress also enhances TH expression in the adrenal gland, which manifests in an increase of NE release in response to stressors in rats [32]. IBS patients with depression display changes in TH gene expression, as well [33]. These findings suggest that some drugs such as reboxetine, which strengthens the adrenergic system may play a role in the treatment of IBS patients with depressive disorders.

### 2.3. Norepinephrine as a Target for Treatment

Preliminary clinical results support a possible therapeutic role for the α2-adrenoreceptor in IBS. A study investigated modifications of NE plasma levels after ingestion of the α2-receptors antagonist, yohimbine (YOH) and agonist clonidine (CLO). The results showed that YOH increased NE plasma levels and anxiety in IBS patients, while CLO decreased NE plasma levels and was associated with more brain activity [30]. Another possible line of treatment focuses on the Corticotrophin Release Factor- Receptor type 1 (CRF-R1). IBS patients present alterations in NE pathways of locus coeruleus complex, and CRF-R1 could attenuate the locus coeruleus complex responsiveness to stressors [34]. The vagus nerve could also be a target for IBS treatment. A vagal reinforcement can be achieved by different techniques as electrical or pharmacological stimulation. Moreover, nonpharmacological approaches such as hypnotherapy or mindfulness seem to increase vagal tone. Visceral pain perception may also be improved by these therapies which reduce epinephrine and TNF-α levels allowing remission maintenance [28].

## 3. Serotonin

Serotonin was previously called enteramin by Erspamer and Asero due to its gastrointestinal functions; after discovering that it was identical to the vasoconstrictor substance known as such, it was renamed serotonin (5-HT, 5-hydroxytriptamin). Serotonin is synthesized from the amino acid tryptophan in enterochromaffin cells from the intestinal epithelium and serotonergic neurons. Ninety-five percent of serotonin production is from the gastrointestinal tract, meanwhile, 5% is from the nervous system. Once in the blood, serotonin can be stored in platelets, in which there are high levels of SERT (serotonin transporter) [35]. SERT uptakes 5-HT into cells, where it can be stored or degraded. SERT function is key to regulate 5-HT’s availability, and consequently 5-HT signalling.

Serotonin has multiple functions at the digestive level as a modulator of gastrointestinal secretion, peristalsis, or absorption; and also at a central level, controlling behaviour and critical neurological functions [36]. Experimental exogenous intake of serotonin results in multiple responses. This wide range of effects is due to the vast localization and diversity of 5-HT receptors [37]. Fourteen different 5-HT receptors have been identified and clustered in seven families based on their signalling pathways. Most of them are coupled to G proteins, and only the 5-HT_3_ receptor is a ligand-gated ion channel [38]. It is now known that some 5-HT receptors have specific functions, although many of them trigger diverse and antagonistic responses [39].

### 3.1. Serotonin’s Role in the Central Nervous System

In the CNS, 5-HT regulates numerous functions such as nociception, motor tone, sleep, sexual behaviour, emesis, and temperature. It also affects vascular tone as a vasoconstrictor molecule, helping other vasoactive mediators as angiotensin II, histamine and NE [40].

Moreover, alterations in the serotonergic system are correlated to some psychiatric diseases such as depression or anxiety disorders. Activity of 5-HT_2C_ receptors seems to increase anxiety [41], and platelet 5-HT levels are increased in patients suffering from anxiety and depression [42]. Almost all serotonin receptors play a role in depression and anxiety-like behaviours. Activation of postsynaptic 5-HT_1A_, 5-HT_1B_, 5-HT_2B_ and 5HT_4_ receptors and inhibition of postsynaptic 5-HT_2A_, 5-HT_3_, 5-HT_5A_ and 5-HT_7_ result in antidepressant-like effects [43].

### 3.2. Serotonin’s Role in the Gastrointestinal System

The release of 5-HT from enterochromaffin cells in the intestinal epithelium occurs mainly after mechanical and chemical stimulus of the intestinal wall when food passes through the intestine [44]. Intestinal microbiota are an essential regulator of 5-HT, as they increase the expression of tryptophan hydroxylase enzymes [7] and regulate serotonin transporter function [45]. 5-HT release can also be regulated by vagal or sympathetic adrenergic stimulation, mucosal changes, obstruction of gut motility or lowering of luminal pH [46]. After its release, 5-HT stimulates the peristaltic reflex, increases ileal and duodenal irrigation and facilitates gastric accommodation mediated by 5-HT_1_, 5-HT_3_, 5-HT_4_ and 5-HT_7_ receptors [47]. To avoid serotonin overstimulation, 5-HT is afterwards taken up by SERT from enterocytes.

There are 3 main 5-HT receptors involved in the regulation of gastrointestinal functions: 5-HT_1_, 5-HT_3_ and 5-HT_4_. Activation of 5-HT_1A_ receptors (mainly localized in submucosally and in the myenteric plexus of the ENS) inhibits ACh release, which leads to a reduction of intestinal smooth muscle contraction–an anticholinergic effect [35]. These receptors are located in the spinal cord as well, where their main function is to reduce somatic pain signalling [48]. 5-HT_3_ receptors (situated in enteric neurons and smooth muscle cells) intervene in the contraction of intestinal smooth muscle (modulating gut motility) and in gut-brain communication through vagal afferent fibres, activating pain-mediating neurons (modulating visceral pain signalling) [49]. The 5-HT_3_ receptor also mediates nociception by activation of inhibitory GABAergic interneurons. Some polymorphisms of this receptor may be associated with IBS-D risk [50]. In fact, the gastrointestinal serotonergic system has been widely associated with some IBS alterations.

Enterochromaffin cells and 5-HT are increased in colonic tissue from IBS rats. In addition, that increment is correlated to higher c-fos levels in CNS. This evidence sustains that CNS activation may induce enterochromaffin cells activation in the colon and subsequent 5-HT release [33]. IBS-D patients show significantly elevated serotonin levels in blood and urine compared with controls and IBS-C patients [51]. However, high serotonin levels do not seem to be specific to an IBS subtype, as other studies have detected increased 5-HT concentrations in both IBS-C and IBS-D patients [52]. IBS patients show lower concentrations of the main 5-HT metabolite, 5-HIAA (5-hydroxyindole acetic acid), and a lower 5-HIAA/5-HT ratio [46], although hypersensitive IBS patients show increased concentrations of 5-HIAA compared with non-hypersensitive ones [53]. A gender influence on 5-HIAA levels and 5-HIAA/5-HT ratio was found in IBS patients, with levels significantly lower in female than male IBS patients. According to IBS subtypes, IBS-M patients displayed the lowest 5-HIAA and 5-HIAA/5-HT ratios compared to IBS-D and IBS-C patients [54]. Moreover, significant differences in the ratio of 5-HIAA/HVA (homovanillic acid, a dopamine metabolite) have been demonstrated among IBS subtypes: IBS-C patients have higher levels of dopamine in plasma and of dopamine metabolites in their urine. 5-HIAA was not the only serotonin metabolite studied, 5-HTP might play a role in hyperalgesia [55]. Lower densities of endocrine cells expressing 5-HT and peptide YY in the colon tissues of IBS patients have been also demonstrated, as well as a reduction of chromogranin A density in the colon of patients with IBS [56].

Some SERT polymorphisms are responsible for pharmacokinetic differences that are observed, for example, in the response of colonic transit to alosetron in IBS-D patients [57]. IBS symptoms including luminal hypersensitivity, augmented peristalsis, or diarrhoea might be explained by changes in SERT expression. Mucosal SERT expression decreases in IBS-C and IBS-D patients. This increases mucosal 5-HT, which could mediate those symptoms [58]. Furthermore, a decrease of mucosal SERT expression is correlated with an increase of mucosal intraepithelial lymphocytes and mast cells in IBS-D patients [59]. The activity of platelet SERT has also been examined with some controversial results. Some studies have shown reduced platelet SERT expression in IBS patients, but other studies have found a reduction only in male IBS patients [60]. Genetic variations in SERT expression are being studied as a possible aetiology for IBS development, hypothesizing a genetic predisposition to IBS. In fact, it was demonstrated that SERT variants could be correlated in IBS patients with psychiatric comorbidities [59]. Interestingly, ethnic differences were found in specific genetic variations. The L/L genotype or the L allele was more frequent in East Asians than in Caucasians with IBS-C, and SLC6A4 polymorphism was found to be associated with a reduced risk of IBS in American and Asian populations [59].

Many studies have described changes in serotonin metabolism in patients with psychiatric comorbidity, but according to Thijssen et al., there is no change in plasma 5-HT metabolites caused by anxiety or depression symptoms [46]. Changes in tryptophan metabolism have been correlated with the manifestation of depressive symptoms in patients with IBS as well. Decreases in kynurenic acid and 5-HT were observed in duodenal mucosa from IBS patients, and these changes were correlated with their psychological state. These data suggest that modulation of the kynurenine/tryptophan pathway influences NMDA receptors in CNS regions involved in the development of depression and may provide useful therapeutic tools to prevent or reduce psychiatric comorbidities of IBS [61].

It must be known that, due to the multifactorial physiopathology of IBS, single-receptor-modulating drugs may not reach enough therapeutic gain. Almost 67% of IBS patients associate their symptoms with diet. A decrease in the intake of foods rich in FODMAPs increases the density of 5-HT and peptide YY in endocrine cells and improves symptoms and quality of life for IBS patients [56]. Other possible diets base on tryptophan modifications have been proposed, as serum tryptophan levels are increased in D-IBS patients compared to healthy controls. However, a dairy-free diet does not change these alterations or eliminate IBS symptoms [62]. Otherwise, kynurenine/tryptophan and melatonin/tryptophan ratios are decreased in IBS-D patients compared to healthy controls, with the latter ratio directly correlated to altered sleep quality in IBS-D patients [63].

### 3.3. The Serotonergic System as a Target for Treatment

Promising IBS management results using agonists and antagonists of 5-HT_3_ and 5-HT_4_ receptors are being explored [64]. Cisapride is a prokinetic drug, a partial 5-HT_4_ receptor agonist, 5-HT_3_ receptor antagonist, and HERG K+ channel blocker. Its effects on smooth gut muscle may be paradoxically due to the blockage of the HERG K+ channel; this action is also the cause of its proarrhythmic effect [65]. Tegaserod is a 5-HT_4_ agonist that is already used to treat IBS-C in women in some parts of the world. As with cisapride, it has prokinetic effects [47] and decreases abdominal contractions during colorectal balloon distension in mice [66]. Other studied 5-HT_4_ agonists are velusetrag and prucalopride which seem to be effective for constipation [67]. Alosetron is approved in the United States for the treatment of female IBS-D patients. It is a 5-HT_3_ receptor antagonist and reduces abdominal pain [68]. It is suggested that alosetron’s effect may occur on the CNS instead of peripherally. PET scans show that alosetron reduces cerebral blood flow in the left anterior insula and inhibits the ventromedial frontal cortex, indicating that alosetron may repress autonomic and emotional processing networks. It was also demonstrated that alosetron and granisetron could cross the blood-brain barrier, supporting the idea that the effect of those drugs is centrally mediated [69]. Granisetron was tested in mice, demonstrating that it could blockade 5-HT-induced hypersensitivity [53]. Ondansetron and Ramosetron are also 5-HT_3_ antagonists. Ramosetron is used in Asia as an antiemetic drug, but it is still not available for therapeutic use in other continents, even though, it is a promising treatment for IBS-D patients, since it improves stool consistency and reduces urgency and frequency of stool [70]. Recently, chanoclavine, a 5-HT_3A_ blocker, has been proposed due to its potential antiemetic effects [71]. 5-HT_1B/D_ receptor agonists such as sumatriptan are also under study. Sumatriptan’s intravenous application delays gastric emptying and causes a significant relaxation of the gastric fundus [72]. In addition to its actions on the upper gastrointestinal tract, sumatriptan also modifies colonic, rectal, and anal sensitivity. Despite its effects on gastric function in dyspeptic patients, sumatriptan and other 5-HT_1_ receptor agonists can have many side effects including constriction of coronary arteries or induction of chest pain by increasing oesophageal visceral sensitivity. Therefore, its daily use may not be possible [73].

Selective serotonin reuptake inhibitors (SSRIs) are a group of antidepressant drugs that include fluoxetine, paroxetine, citalopram, and sertraline, among others. Their increase on serotonergic activity is mainly due to SERT inhibition. There are contradictory studies about the effects of those drugs on 5-HT plasma levels in IBS patients. Some studies affirm that administration of citalopram in IBS patients leads to an increase of 5-HT plasma levels, but it is still unknown how this increase may change 5-HT activity at the CNS or intestinal levels [74]. There is conflicting information about the use of antidepressants to treat functional gastrointestinal disorders [75]. Although some of them improve IBS symptoms, their side effects can reduce their applicability to treat IBS. Actually, venlafaxine (a serotonin-NE reuptake inhibitor) improves gastric and colonic symptoms but can also cause fatigue, hypertension or dyslipidemia [75]. Moreover, there is some controversy also exists regarding citalopram’s efficacy in treating IBS symptoms compared to placebo [76,77]. Other SSRIs have been studied with diverse results. Paroxetine enhanced patients’ perception of well-being, but did not ameliorate abdominal symptoms [78]. In contrast, some studies found that fluoxetine improves abdominal symptoms and stool frequency in IBS-C patients [79]. Patients treated with low doses of amitriptyline (a tricyclic antidepressant) reported amelioration of all symptoms [80]. All these data suggest that SSRIs could be a useful treatment for IBS, but more clinical trials and studies are needed to clarify the controversial results [81].

## 4. Glutamate

Glutamate is the main excitatory neurotransmitter in the CNS [82], and it has been described as having multiple roles as a nutrient, catalytic intermediate, or excitatory molecule [83]. Glutamate is an amino acid that can be introduced exogenously through the diet; however, exogenous glutamate crosses neither the intestinal barrier nor blood-brain barrier. Glutamate as a neurotransmitter is produced *de novo* in the brain from glucose [84]. After glutamate release from neurons, this amino acid is taken up by glia cells, and there, it is turned to glutamine by glutamine-synthetase for recycling to neurons. Glutamate reserves are refilled again when glutamine is engrossed by neurons. This means glutamine metabolism is the principal cycle for replacement of glutamate in neurons [85]. A high protein diet decreases glutamate and glutamine concentrations in plasma, although this phenomenon is still unexplained [86].

### 4.1. Glutamate in the Central Nervous System

In the CNS, glutamate plays a role in learning, motor activity, memory, neural development and synaptic plasticity [84]. Its involvement in processing pain was demonstrated through the measurement of glutamate levels in cerebrospinal fluid because higher levels of glutamate are correlated to heavy pain [87].

Glutamate receptors are divided into ionotropic glutamate receptors (iGluRs) and metabotropic glutamate receptors (mGluRs). In turn, mGluRs are clustered in 3 groups: I, II, and III [88]. Receptors belonging to groups II and III act as regulators, inhibiting glutamate release [89]. Eight different mGluR subtypes (mGluR1 to mGluR8) exist. The first studies of glutamate supported the theory that its receptors were in the CNS, but recent results confirmed that mGluRs are also expressed peripherally, such as in the gastrointestinal system [89]. IGluRs are divided into 3 subtypes: N-methyl-D-aspartate (NMDA), amino-3-hydroxy-5-methyl-4-isoxazole propionate, and kainate receptors. Located in the esophagus, NMDA receptors are involved in the process of swallowing [90]. Depression and anxiety disorders have been associated with glutamatergic changes, especially in mGluRs, because effective antidepressants activate this group of glutamate receptors [91].

### 4.2. Glutamate’s Role in the Gastrointestinal Tract

Glutamate modulates energy metabolism in the gastrointestinal system at pre and postprandial phases. Glutamate also seems to enhance digestion and nutrient absorption via brain activation by the vagus nerve [92]. It is conjectured that the glutamate receptor located in the stomach is mGluR1, and that its role is to stimulate 5-HT release indirectly through excitation of vagal afferents. On the other hand, glutamate decreases somatostatin release, stimulating exocrine and endocrine functions in the GI tract [93]. The activation of mGluR7 increases colonic secretory function, while mGluR8 plays a role in colon motility. Moreover, mGluR7 could be associated with IBS, because its expression is increased in colon of rats with visceral hypersensitivity [94]. Glutamate injection into the stomach, duodenum, and portal vein results in the activation of afferent fibres on the gastric, celiac and hepato-portal vagal branches. This activation in the stomach seems to stem from the vagus nerve via 5-HT receptors. In fact, granisetron, a selective inhibitor of the 5-HT_3_ receptor, can inhibit this response [95].

IBS patients show reduced glutamate and glutamine concentrations, although glutamine was disjointed to psychological or gastrointestinal symptoms [96]. These results are contradictory regarding pain because glutamate concentrations are elevated in fibromyalgia and chronic pelvic pain [97]. Oppositely, lower concentrations of glutamine can be a predictor of the duration of abdominal pain in IBS patients [98]. Lower glutamate levels and disruptive glutamate receptors expression could point to glutamate as a possible therapeutic target for IBS. In fact, AMN082, a mGluR7 agonist, showed a decrease in colorectal distension-induced visceral hypersensitivity and a reduction in the inflammatory response via inhibition of NF-κB in IBS rats [99]. An anxiolytic effect has also been described in the CNS, modulating GABAergic neurotransmission [100].

Central changes in the glutamatergic system in relation to visceral hypersensitivity have been studied in animals, showing that rats suffering from induced colitis and visceral pain manifested increased levels of GluN2B and GluA2 receptors in the anterior cingulate cortex [101].

### 4.3. Glutamate as a Target for Treatment

Changes in dietary glutamate have also been studied for the management of IBS and fibromyalgia. A glutamate-rich diet worsens IBS and fibromyalgia symptoms. Although different doses of glutamate as nutritional supplement have been investigated for the treatment of dyspepsia, functional dyspepsia, gastrointestinal ulcer, and diarrhoea with improvement of symptoms [95], higher dietary glutamate levels have been associated with abdominal bloating, diarrhoea, and abdominal pain [102]. However, glutamine supplementation seems to be beneficial in some cases. Actually, in IBS-D patients with intestinal hyperpermeability following an enteric infection, oral dietary glutamine supplements dramatically and safely reduced all major IBS-related endpoints [103].

mGluR5 has been found peripherally in the gastrointestinal tract. After this discovery, several trials have emerged targeting those receptors. mGluR5 antagonists such as MPEP or SIB1893 remove IL-1β-induced mechanical allodynia in rats [104]. MPEP also diminishes reflux symptoms by inhibiting the transient lower sphincter relaxation. Patients with gastroesophageal reflux disease reported improvement in acid reflux with the use of ADX10059 (a mGluR5 negative allosteric modulator) [104]. Moreover, glutamate uptake activators such as riluzole seem to improve visceral hypersensitivity in stressed animals, having no effect on naive rats [105]. Nausea and emesis could be treated by the blockade of non-NMDA iGluRs. In fact, NBQX eliminates salivary secretion and nausea [90]. On the other hand, antagonists of NMDA receptors could be beneficial for visceral pain, which was shown in male mice faced with the hot plate and writhing tests [106]. Despite their useful pharmacological applications, iGluRs modulators cannot be used as long-term treatment due to their psychiatric side effects [105].

## 5. Gamma-Aminobutyric ACID

Gamma-aminobutyric acid (GABA) is an amino acid derivate of glutamate. Glutamic acid decarboxylase (GAD) enzyme is responsible for the conversion of glutamate to GABA by α-decarboxylation; afterwards, GAD interacts with the vesicular GABA transporter mediating the vesicular uptake of GABA [105]. Brain-derived neurotrophic factor (BDNF) increases GAD expression, regulating GABA homeostasis [106]. Ninety percent of the GABA synthesized is subsequently degraded by GABA-transaminase, which is present in neurons and glia cells. After its release from the nervous system, GABA transporter uptakes GABA from the synaptic cleft.

### 5.1. GABA in the Central Nervous System

GABA is the primary inhibitory neurotransmitter in the CNS [107]. Its inhibitory function is shared with the neurotransmitter glycine in the mammalian CNS [108]. It functions to reduce neuronal excitability by inhibiting nerve transmission. GABAergic neurons are located in the hippocampus, thalamus, basal ganglia, hypothalamus, and brainstem. The balance between inhibitory neuronal transmission via GABA and excitatory neuronal transmission via glutamate is essential for proper cell membrane stability and neurologic function. GABA is conjectured to have effects on motor performance and cognitive functioning because a decrease of GABA levels in elderly patients seems to be associated to the deterioration of these abilities [109]. It also plays a role as a source of energy, generating ATP in the tricarboxylic acid cycle in the mitochondria [110]. There are 2 main types of GABA receptors: GABA-A (fast-acting ionotropic receptors) and GABA-B (slower-acting metabotropic receptors) [110]. GABA-A receptors are divided into 19 subunits that can be located in neuronal and nonneuronal cells [111]. They are chlorine ion channels, whereas GABA-B receptors are G-protein coupled receptors [108]. GABA-A receptors are localized in synaptic and extrasynaptic sites. Synaptic sites mediate phasic inhibition and extrasynaptic ones mediate tonic inhibition [112]. Non-neuronal GABA- receptors play a role in fluid secretion in lungs and intestine [113] while in central nervous system GABA can play the role of gliotransmitter when it is released from astrocytes [114]. Recent studies have also described a third GABA-receptor: GABA-ρ or GABA-C receptor. This receptor is also considered a subtype of GABA-A receptor, which is mainly localized in the eye and involved in visual image processing [115]. Another receptor that GABA shares with glutamate is GAT, which mediates the uptake of both neurotransmitters. GAT is present in glial cells and neurons. Four types of GAT transport GABA: GAT1, GAT2, GAT3, and BGT-1. GABA transporters function by the gradients of Na^+^ and Cl^−^. GAT1 is the major GABA transporter, and it is mainly localized in the cerebral cortex, whereas GAT3 is found in the brainstem. Otherwise, GAT2 is expressed in liver and kidney, and to a lesser extent in the leptomeninges [116]. Because GABA is an inhibitory neurotransmitter, decreasing its concentration would produce a feeling of anxiety. It has also been associated with schizophrenia, autism spectrum disorder, and major depressive disorder.

### 5.2. GABA’s Role in the Gastrointestinal Tract

In the gastrointestinal tract, GABA has multiple functions such as visceral nociception, modulation of colonic afferent excitability, gastrointestinal secretion, and motility or enhancement of the local immune system [117]. The different GAT isoforms are present in the gastrointestinal tract: GAT2 is predominantly localized in enteric glia cells and GAT3 in myenteric neurons [118]. A GABAergic signal system in the intestinal epithelial cells has been demonstrated and has a role in the pathogenesis of allergic diarrhoea by activation of submucosal secretomotor neurons [113].

As the main inhibitory neurotransmitter, GABA plays a protective role in inflammatory diseases by modulating the production of cytokines. Actually, GABA levels are decreased in serum samples of patients suffering from multiple sclerosis, ischaemic stroke, ulcerative colitis, and other inflammatory diseases [119]. The GABAergic system is also altered in IBS patients. IBS-D patients show diminished levels of GABA, GAD2, and GABA- B receptors subtype B1 and B2, as well as increased GAT-2 [119]. Not only are GABA-B receptors altered in IBS patients, but Selfi et al. have also demonstrated higher levels of GABA-A receptor α3 in colon from mice exposed to stress, showing that stress could be responsible for GABAergic alteration in IBS.

Hypersensitivity to visceral pain is a key IBS symptom. In this line, patients suffering from chronic pelvic pain had lower levels of GABA in anterior cingulate cortex [120]. Moreover, anxiety disorders are comorbid pathologies highly related to IBS. GABA levels in the prefrontal cortex appear to be increased in IBS patients with highly severe anxiety symptoms, but not in IBS patients without comorbid anxiety disorders. However, these GABAergic alterations are not related to gastrointestinal symptoms, pain or depression [96].

### 5.3. GABA as a Target for Treatment

GABA agonists or analogues such as pregabalin or gabapentin could be useful for IBS treatment. As Zhang et al. proved, gabapentin improves pain and anxiety-like behaviours in mice, although the pharmacological use of this drug for the treatment of IBS should be limited due to its serious side effects (hepatotoxicity and neurotoxicity) [121]. Gabapentin also demonstrated a reduction in the cerebral nociceptive response to colorectal distension. The FDA has approved pregabaline for the treatment of fibromyalgia and neuropathic pain for its analgesic and anxiolytic effects [122]. In IBS-D and IBS-M, pregabalin seems to improve abdominal pain, diarrhoea, and bloating, but it did not affect the quality of life, anxiety or depression, and IBS symptoms in IBS-C patients [122]. The improvement of pregabalin in IBS symptoms may be explained by its binding to calcium channels of the enteric neurons in the ileum [123]. The use of baclofen (a GABA-B receptor agonist) and gabapentin has been investigated to reduce visceral sensitivity in rats. Baclofen can decrease visceromotor response, but its effect does not seem to be significant and its side effects do not allow its use as chronic treatment [124]. CGP7930 is another GABA-BR agonist that can reduce visceral pain without as many side effects as baclofen due to its mechanism of action, that enhances endogenous GABA release [118]. Despite being a promising target for IBS treatment, activation of GABA-A receptors has also shown important side effects such as exacerbation of acute colitis [125]. Other possible alternative treatments for GABA-dependent gastrointestinal symptoms are the use of genetically modified GAD-productor *Bifidobacterium longum* [126] or GABA containing functional foods such as enriched goat milk [127].

## 6. Acetylcholine

Ach is an excitatory neurotransmitter that is named after its chemical structure consisting of acetic acid and choline. Choline is present in dietary foods, and acetic acid derives from mitochondrial coenzyme acetyl-coA. The synthesis of ACh takes place in axon terminals and is catalysed by the enzyme choline-acetyl-transferase; then it is introduced in synaptic vesicles by the vesicular ACh transporter. After its release and binding to nicotinic or muscarinic receptors, ACh is degraded by acetylcholinesterase, mainly present in the synaptic cleft. Once hydrolysed, choline returns to presynaptic neurons by the action of a high-affinity choline transporter.

### 6.1. Acetylcholine’s in the Central Nervous System

ACh acts at various sites within the CNS, where it can function as a neurotransmitter and as a neuromodulator. It plays a role in motivation, arousal, attention, learning, and memory, and is involved in promoting REM sleep. ACh signalling can be mediated by nicotinic and muscarinic receptors; nicotinic receptors are ion channel ligated, whereas muscarinic ones are ligated to G proteins. Nicotinic receptors are composed of 5 homologous subunits, but those localized in neuromuscular junctions consist of different subunits that are different from neuronal ones. Although activation of nicotinic receptors shows variable responses depending on the subunit composition, their activation usually produces membrane depolarization [128]. Among their functions, these receptors play a role in enhancing neuromodulation and release of different neurotransmitters such as glutamate and GABA [129]. They are especially vulnerable to the deposit of ß-amyloid peptide in the pathogenesis of Alzheimer’s disease, manifesting a down-regulation of these receptors in Alzheimer’s patients [129]. On the other hand, there are 5 subtypes of muscarinic receptors: M1, M2, M3, M4, and M5 that can be classified into 2 groups, depending on their associated G protein: M1, M3, and M5 are ligated to the family of G_q/11_ proteins [130] and their activation increases neuronal excitability [131], whereas M2 and M4 are joined to G_i/o_-type G proteins [130] and their activation produces postsynaptic inhibition [131]. Muscarinic receptors are involved in memory, motor function and learning; in fact, M1 is associated with cognitive processing, memory, and learning. M2 expression is decreased in patients with Alzheimer’s disease and associated with the neuropsychiatric behaviour of these patients [132].

ACh is known for its function as a key neurotransmitter and mediator of the communication between neurons and muscle cells, but it also plays an important role in the autonomous nervous system regulating heart rate, digestion, breathing, or vasodilation [133]. The release of ACh in neocortical cells is associated with a state of vigilance, but in contrast, Ach can also be key in sleep phases. The participation of ACh in cognitive function and episodic as well as semantic memory is also recognized, especially in the hippocampus. In both sites (hippocampus and neocortex), ACh enhances experience-dependent plasticity in synergic action with NE [134]. In addition, ACh plays an important role as a neuromodulator, enhancing neuronal responses to internal and external stimuli [133] Actually, ACh enhances T cell migration into infected tissues in the immune response [135].

ACh’s functions mostly depend on its concentration. ACh concentration oscillates with circadian rhythms, but other stimuli (as caffein or attentional demands) may trigger variations in the concentration of ACh [136]. Alterations in the cholinergic system are associated with the pathogenesis of different mental pathologies such as schizophrenia, major depression, or bipolar disorder. In fact, ACh receptor antagonists and inhibitors of acetylcholinesterase are used for the treatment of depressive symptoms and visual hallucinations [129].

### 6.2. Acetylcholine’s Role in the Gastrointestinal System

In the gastrointestinal system, ACh is involved in colonic motility [137]. Higher levels of ACh result in an increase in gastrointestinal motility [138], but a decrease in cholinergic function in the elderly may explain their propensity to have constipation [137]. ACh also modulates Cl^-^ secretion, mainly via M3 muscarinic receptors and to a lesser extent M1 muscarinic receptors as well [139]. ACh is released from vagal efferent nerves then it joins α7 nicotinic ACh receptors, inhibiting TNFα from macrophages and decreasing intestinal permeability. Moreover, it is conjectured that vagus nerve stimulation by those receptors could mediate a protective role in the intestinal epithelium barrier [140].

ACh may be involved in IBS pathophysiology, because IBS’s comorbidities (especially anxiety disorders and stress) produce changes in ACh levels. Acute stress suppresses ACh synthesis in the intestine and brain by inhibiting the production of choline acetyltransferase, and favouring the synthesis of acetylcholinesterase [141], which is associated with an inflammatory effect due to the loss of inflammatory inhibition mediated by ACh [142]. In this context, some studies have shown that acute stress in maternally separated rats with IBS results in increased colonic motility, mediated by ACh [143]. This may be clinically translated into augmentation of stool frequency. Furthermore, blocking muscarinic receptors with atropine inhibits stress-induced diarrhea [144]. In contrast, IBS-C patients showed no differences in the secretory response of colonic mucosa to acetylcholine [145]. Because it is recognized that the development of IBS and other gastrointestinal diseases is joined to early life stress, changes in the cholinergic system were studied in pigs exposed to early weaning stress. Compared to controls, an upregulation of the cholinergic activity in early weaning stress pigs was expressed as the absence of decrease of ChAT neurons in the GI tract [146].

### 6.3. Acetylcholine as a Target for Treatment

Mediators of colonic mucosa in IBS patients have been demonstrated to activate ACh release from myenteric neurons via mast cells independently of the bowel habit [147]. Hyperalgesia and visceral hypersensitivity have been associated with increased expression of high-affinity choline transporter (HAChT) [148]. This augmentation can result in an increase in ACh levels, which has an antinociceptive role [149]. Pharmacological modifications of the upregulation of HAChT have been done with ammonium pyrrolidinedithiocarbamate, which abolishes this phenomenon. Moreover, MKC-231 can enhance HAChT activity, resulting in a decrease in visceral pain [150]. Most of the investigated drugs with cholinergic effects in the treatment of IBS reduce colonic motility and stool frequency. Muscarinic antagonists (e.g., dicyclomine) inhibit colonic contractility, which can be an effective way to manage symptoms such as abdominal pain and diarrhoea [151]. In Australia, the utilization of mebeverine is approved for the treatment of alterations in the bowel transit and abdominal pain. Mebeverine acts as an antagonist of muscarinic receptors and an inhibitor of NE uptake [152]. Pinaverium is also prescribed for the same gastrointestinal symptoms, because its anticholinergic effect only takes place on smooth intestinal muscle, reducing systemic effects [153]. Recent studies have demonstrated the potential use of selective M3-antagonists. Darifenacin may regulate gastrointestinal motility in a manner more pronounced than is seen in non-selective antagonists such as tolterodine. In fact, in patients with IBS-D, darifenacin causes a significant delay of intestinal and colonic transit compared to alosetron [154]. Tolterodine is nowadays used for the treatment of overactive bladder. As a muscarinic receptor antagonist, one of its main side effects is constipation, although it is proven that no differences in bowel transit occur with placebo [155]. Moreover, anticholinergic drugs used for the treatment of overactive bladder were tested in different intestinal diseases resulting in an improvement of IBS symptoms [156]. Another muscarinic antagonist used for the treatment of IBS is zamifenacin (a partially selected M3 antagonist), which reduces postpandrial colonic contractility [157]. Apart from the use and research of drugs acting on muscarinic receptors, other drugs that target different receptors have also been studied. Cannabinoid receptors are in cholinergic neurons; thus, cannabinoid agonists also have cholinergic effects. Dronabinol (a cannabinoid receptor agonist) has been probed in IBS patients, showing a reduction in gastrointestinal motility and gain in colonic compliance in IBS-D and IBS-M subtypes but not in IBS-C [158]. In addition, some serotonin antagonists also have anticholinergic effects, including alosetron, ramosetron, cilansetron, ondansetron, and granisetron; they can reduce gastrointestinal peristalsis and upgrade abdominal pain [159].

## 7. Other Neurotransmitters

Here we have explored the role of main neurotransmitters in IBS, but the involvement of other neurotransmitters cannot be neglected. Several studies have pointed out the potential role of histamine and dopamine in IBS pathogenesis.

Histamine has been related to gastrointestinal inflammation and abdominal pain. The main histamine receptors, which take part in gastrointestinal processes, are H1 and H4, although H2 is related to the production of gastric acid [160]. In IBS patients, levels of urinary histamine have correlated to the severity of IBS symptoms, especially abdominal pain [161]. The administration of an H1-antagonist revealed different responses in IBS patients compared to healthy controls, demonstrating possible overstimulation of the histaminergic system in IBS patients [162]. H1 and H4 receptors could have a key role in the pathogenesis of colitis and postinflammatory visceral hypersensitivity, because their expression is increased in colon tissue of rats that have colitis. JNJ7777120, an H4-antagonist, seemed to ameliorate abdominal pain in that postinflammatory colitis model [163]. Novel interventions are being proposed that involve blocking H1 receptors, as ebastine has been found to improve IBS symptoms, including visceral hypersensitivity and abdominal pain [164], and ketotifen has been found to enhance health-related quality of life and increase the pain threshold in IBS patients [165]. Similarly, AST-12O, which adsorbs histamine from the intestinal lumen, could reduce pain and bloating in IBS-D and IBS-M patients [166].

On the other hand, several studies have investigated alterations in the dopaminergic system in IBS patients. In fact, IBS patients show lower dopamine levels in plasma [51] and urine [161] compared to healthy controls. Dopamine mediates colonic peristalsis, activating muscle contraction through D1 receptors and inhibiting it by D2 receptors [167], being related to motility dysfunction. However, the administration of dopamine or its agonists enhances IBS symptoms in patients with comorbid restless legs syndrome [168]. Nowadays, metformin is a widely used drug for the treatment of mellitus diabetes type II. Nevertheless, this drug has been studied for its antinociceptive effect through the activation of central D2 dopamine receptors in IBS patients [169]. Similarly, activation of those dopaminergic receptors by butyrate enemas decreases visceral allodynia and colonic hyperpermeability [170].

## 8. Conclusions

Managing IBS has attracted major attention because single-agent therapy rarely relieves bothersome symptoms for all patients. In clinical practice, there is still a lack of effective treatment for IBS, and the prescribed drugs usually alleviate only one symptom of the whole syndrome. IBS patients display some neurotransmitter dysfunctions that could cause disruption of gut homeostasis and the onset of gastrointestinal symptoms such as abdominal pain, bloating and changes in stool frequency in IBS. A more exhaustive personalized analysis in relation to neurotransmitters in IBS patients would be necessary to develop strategies that are more effective and achieve a better understanding of the role of the gut-brain axis in the pathogenesis of the syndrome.

Here, we have evaluated the current evidence of neurotransmitter dysfunction in IBS and explored its potential therapeutic use. Dysfunctions of key neurotransmitters such as norepinephrine, serotonin, glutamate, GABA, or acetylcholine could help to understand IBS pathophysiology and open the door of new approaches for IBS management.

Some drugs focused on neurotransmitters are being explored for the management of IBS symptoms (Table 1), however, the interaction between different neurotransmitters should be considered. Even if evidence of improvement of IBS symptoms exists, new targets and therapies are needed. In this context, finding novel targets for specific neurotransmitters’ receptors to reduce side effects is critical. The use of antidepressants for the treatment of IBS is controversial due to their adverse effects. SSRIs improve psychological symptoms in depressive and anxiety disorders, but their effect on gastrointestinal symptoms is limited. Individualized treatment could be an alternative for patients with comorbid anxiety or depressive disorders. The development of more selective molecules as isoform-targeted agonists and antagonists of serotonin receptors [171] would provide novel approaches with minimal side effects. In addition, we cannot forget the effect of diet on the production and metabolism of neurotransmitters [172].

Finally, numerous pieces of evidence suggest that changes in the microbiota are correlated with the development of visceral hypersensitivity, which represents one of the major symptoms in IBS patients [172]. Recent studies have demonstrated the crucial inter-relationship between bacteria and neurotransmitters. Gut microbiota can produce neurotransmitters, modulate host production and even regulate their signalling. Therefore, more studies addressing the microbiota-gut-brain axis in the IBS context are needed. An innovative and intriguing approach has been opened by the possibility of modulating neurotransmitter signalling along the microbiota-gut-brain axis by influencing the microbiota composition [61]. Microbiota modulation by probiotics, prebiotics or faecal transplantation could bring new approaches for IBS management. In fact, a randomized, double-blind, placebo-controlled trial showed improved diversity of microbiota in faecal transplantation IBD patients [173]. Promising results concerning probiotics as a new approach to IBS have also been obtained [173]. Research in this field opens an exciting scenario on the possibility of targeting neurotransmitter signalling, by means of traditional pharmacological approaches as well as by microbiota modulation as new potentially therapeutic tools addressed to irritable bowel syndrome.

## Figures and Tables

**Table 1:** Summary of the drugs targeting neurotransmitters used in IBS.

Neurotransmitter	Drug	Receptor	Effect	Pharmacological Use	References
**SEROTONIN**	CISAPRIDE	5-HT_4_ agonist and 5-HT_3_ antagonist	Prokinetic	Use for the treatment of Gastroesophageal reflux, functional dyspepsia and gastroparesis	Pytliak et al. 2011 [65]
	TEGASEROD	5-HT_4_ agonist	Prokinetic	Use for the treatment of IBS-C	Crowell et al. 2001 [66]
	VELUSETRAG	5-HT_4_ agonist	Prokinetic	Clinical trials have to be done for its approvement	Terry et al. 2017 [67]
	PRUCALOPRIDE	5-HT_4_ agonist	Prokinetic	Used for the treatment of IBS-C	Terry et al. 2017 [67]
	ALOSETRON	5-HT_3_ antagonist	Decreases GI motility	Approved in the USA for the treatment of IBS-D	Lacy et al. 2018 [68]
	ONDASETRON	5-HT_3_ antagonist	Antiemetic, it reduces abdominal pain	Used as antiemetic	Min et al. 2015 [70]
	RAMOSETRON	5-HT_3_ antagonist	Antiemetic	Used as antiemetic in Asia	Min et al. 2015 [70]
	SUMATRIPTAN	5-HT_1B/D_ agonist	Delays gastric emptying	Many side effects to be approved	Mulak et al. 2006 [73]
**GABA**	PREGABALIN	GABA analogous	Analgesic and anxiolytic	Use for the treatment of neuropathic pain	Saito et al. 2019 [122]
	GABAPENTIN	GABA analogous	Analgesic and anxiolytic	Use for the treatment of neuropathic pain	Zhang et al. 2014 [121]
	CGP7930	GABA-B receptor agonist	Reduces visceral pain	Clinical trials have to be done for its approvement	Hyland et al. 2010 [118]
	BACLOFEN	GABA-B receptor agonist	Reduces visceromotor response	Use for the treatment of spasticity and muscle spasms	Nissen et al. 2018 [124]
**GLUTAMATE**	RILUZOLE	Glutamate reuptake activator	Improves visceral hypersensitivity	Use for the treatment of Amyotrophic Lateral Sclerosis	Moloney et al. 2015 [105]
	MPEP	mGluR5 antagonist	Reduces allodynia	Clinical trials have to be done for its approvement	Ferrigno et al. 2017 [104]
	AMN082	mGluR7 agonist	Reduces visceral hypersensitivitiy induced by colorectal distension	Clinical trials have to be done for its approvement	Shao et al. 2019 [99]
**ACETYLCHOLINE**	ZAMIFENACIN	Partially selected muscarinic M3 antagonist	Decreases colonic contractility	Clinical trials have to be done for its approvement	Houghton et al. 1997 [157]
	TOLTERODINE	Non-selective muscarinic antagonist	Induces constipation	Use for the treatment of overactive bladder syndrome	Bharucha et al. 2008 [155]
	MEBEVERINE	Muscarinic antagonist	Improves bowel transit and abdominal pain	Approved in Australia for IBS treatment	Dumitrascu et al. 2014 [152]
	DARIFENACIN	M3 antagonist	Improves IBS bowel habits	Use for the treatment of overactive bladder syndrome	De Schryver et al. 2000 [154]
	PINAVERIUM	Anticholinergic effect	Antispasmodic	Approved for the treatment of functional gastrointestinal diseases, as IBS	Zheng et al. 2015 [153]

## References

[1] Drossman D.A., Hasler W.L. (2016). Rome IV-Functional GI Disorders: Disorders of Gut-Brain Interaction. Gastroenterology.

[2] El-Salhy M. (2012). Irritable bowel syndrome: Diagnosis and pathogenesis. World J. Gastroenterol..

[3] Tanaka Y., Kanazawa M., Kano M., Tashiro M., Fukudo S. (2018). Relationship between sympathoadrenal and pituitary-adrenal response during colorectal distention in the presence of corticotropin- releasing hormone in patients with irritable bowel syndrome and healthy controls. PLoS ONE.

[4] Mayer A.E., Labus J., Aziz Q., Tracey I., Kilpatrick L., Elsenbruch S., Schweinhardt P., Van Oudenhove L., Borsook D. (2019). Role of brain imaging in disorders of brain-gut interaction: A Rome Working Team Report. Gut.

[5] Surdea-Blaga T., Băban A., Dumitrascu D.L. (2012). Psychosocial determinants of irritable bowel syndrome. World J. Gastroenterol..

[6] Wang H.X., Wang Y.P. (2016). Gut Microbiota-brain Axis. Chin. Med. J. (Engl.).

[7] Labus J.S., Osadchiy V., Hsiao E.Y., Tap J., Derrien M., Gupta A., Tillisch K., Le Nevé B., Grinsvall C., Ljungberg M. (2019). Evidence for an association of gut microbial Clostridia with brain functional connectivity and gastrointestinal sensorimotor function in patients with irritable bowel syndrome, based on tripartite network analysis. Microbiome.

[8] Husebye E., Hellstrom P., Sundler F., Chen J., Midtvedt T. (2001). Influence of microbial species on small intestinal myoelectric activity and transit in germ-free rats. Am. J. Physiol. Gastrointest. Liver Physiol..

[9] Simrén M., Barbara G., Flint H.J., Spiegel B.M., Spiller R.C., Vanner S., Verdu E.F., Whorwell P.J., Zoetendal E.G., Rome Foundation Committee (2013). Intestinal microbiota in functional bowel disorders: A Rome foundation report. Gut.

[10] Rajilić-Stojanović M., Jonkers D.M., Salonen A., Hanevik K., Raes J., Jalanka J., de Vos W.M., Manichanh C., Golic N., Enck P. (2015). Intestinal Microbiota And Diet in IBS: Causes, Consequences, or Epiphenomena?. Am. J. Gastroenterol..

[11] Ford A., Sperber A., Corsetti M., Camilleri M. (2020). Irritable bowel syndrome. Lancet.

[12] Borodovitsyna O., Flamini M., Chandler D. (2017). Noradrenergic Modulation of Cognition in Health and Disease. Neural Plast..

[13] Clark K.L., Noudoost B. (2014). The role of prefrontal catecholamines in attention and working memory. Front. Neural Circuits.

[14] Alcántara-Hernández R., Hernández-Méndez A. (2018). Complejos moleculares de la señalización adrenérgica [Adrenergic signaling molecular complexes]. Gac. Med. Mex..

[15] Ramos B.P., Arnsten A.F. (2007). Adrenergic pharmacology and cognition: Focus on the prefrontal cortex. Pharmacol. Ther..

[16] Griffen T.C., Maffei A. (2014). GABAergic synapses: Their plasticity and role in sensory cortex. Front. Cell. Neurosci..

[17] Alhayek S., Preuss C.V. (2021). Beta 1 Receptors. StatPearls [Internet].

[18] Choudhury B.K., Shi X.Z., Sarna S.K. (2009). Norepinephrine mediates the transcriptional effects of heterotypic chronic stress on colonic motor function. Am. J. Physiol. Gastrointest. Liver Physiol..

[19] Zou N., Lv H., Li J., Yang N., Xue H., Zhu J., Qian J. (2008). Changes in brain G proteins and colonic sympathetic neural signaling in chronic-acute combined stress rat model of irritable bowel syndrome (IBS). Transl. Res..

[20] Kim H.J., Camilleri M., Carlson P.J., Cremonini F., Ferber I., Stephens D., McKinzie S., Zinsmeister A.R., Urrutia R. (2004). Association of distinct alpha(2) adrenoceptor and serotonin transporter polymorphisms with constipation and somatic symptoms in functional gastrointestinal disorders. Gut.

[21] Naitou K., Shiina T., Kato K., Nakamori H., Sano Y., Shimizu Y. (2015). Colokinetic effect of noradrenaline in the spinal defecation center: Implication for motility disorders. Sci. Rep..

[22] Jubelin G., Desvaux M., Schüller S., Etienne-Mesmin L., Muniesa M., Blanquet-Diot S. (2018). Modulation of Enterohaemorrhagic Escherichia coli Survival and Virulence in the Human Gastrointestinal Tract. Microorganisms.

[23] Strandwitz P. (2018). Neurotransmitter modulation by the gut microbiota. Brain Res..

[24] Asano Y., Hiramoto T., Nishino R., Aiba Y., Kimura T., Yoshihara K., Koga Y., Sudo N. (2012). Critical role of gut microbiota in the production of biologically active, free catecholamines in the gut lumen of mice. Am. J. Physiol. Gastrointest. Liver Physiol..

[25] Carrasco G.A., Van De Kar L.D. (2003). Neuroendocrine pharmacology of stress. Eur. J. Pharmacol..

[26] Deechakawan W., Heitkemper M., Cain K., Burr R., Jarrett M. (2014). Anxiety, depression, and catecholamine levels after self-management intervention in irritable bowel syndrome. Gastroenterol. Nurs..

[27] Burr R.L., Jarrett M.E., Cain K.C., Jun S., Heitkemper M.M. (2010). Catecholamine and Cortisol Levels during Sleep in Women with Irritable Bowel Syndrome. Neurogastroenterol. Motil..

[28] Pellissier S., Dantzer C., Mondillon L., Trocme C., Gauchez A.S., Ducros V., Mathieu N., Toussaint B., Fournier A., Canini F. (2014). Relationship between vagal tone, cortisol, TNF-alpha, epinephrine and negative affects in Crohn’s disease and irritable bowel syndrome. PLoS ONE.

[29] Elsenbruch S., Holtmann G., Oezcan D., Lysson A., Janssen O., Goebel M.U., Schedlowski M. (2004). Are there alterations of neuroendocrine and cellular immune responses to nutrients in women with irritable bowel syndrome?. Am. J. Gastroenterol..

[30] Berman S., Suyenobua B., Naliboff B.D., Bueller J., Stains J., Wong H., Mandelkern M., Fitzgerald L., Ohning G., Gupta A. (2012). Evidence for alterations in central noradrenergic signaling in irritable bowel syndrome. Neuroimage.

[31] Toral M., Robles-Vera I., De La Visitación N., Romero M., Yang T., Sánchez M., Gómez-Guzmán M., Jiménez R., Raizada M.K., Duarte J. (2019). Critical Role of the Interaction Gut Microbiota—Sympathetic Nervous System in the Regulation of Blood Pressure. Front. Physiol..

[32] Miner L.H., Jedema H.P., Moore F.W., Blakely R.D., Grace A.A., Susan R. (2006). Chronic stress increases the plasmalemmal distribution of the norepinephrine transporter and the coexpression of tyrosine hydroxylase in norepinephrine axons in the prefrontal cortex. J. Neurosci..

[33] Zhang R., Zou N., Li J., Lv H., Wei J., Fang X.C., Qian J.M. (2011). Elevated expression of c-fos in central nervous system correlates with visceral hypersensitivity in irritable bowel syndrome (IBS): A new target for IBS treatment. Int. J. Color Dis..

[34] Hubbard C.S., Labus J.S., Bueller J., Stains J., Suyenobu B., Dukes G.E., Kelleher D.L., Tillisch K., Naliboff B.D., Mayer E.A. (2011). Corticotropin-releasing factor receptor 1 antagonist alters regional activation and effective connectivity in an emotional-arousal circuit during expectation of abdominal pain. J. Neurosci..

[35] Sebastián J.J., Sebastián B. (2018). Serotonin and the two brains: Conductor of orchestra of intestinal physiology and mood role in irritable bowel syndrome. Med. Nat..

[36] Spohn S.N., Mawe G.M. (2017). Non-conventional features of peripheral serotonin signaling Stephanie. Nat. Rev. Gastroenterol. Hepatol..

[37] Sharp T., Barnes N.M. (2020). Central 5-HT receptors and their function; present and future. Neuropharmacology.

[38] Göthert M. (2013). Serotonin discovery and stepwise disclosure of 5-HT receptor complexity over four decades. Part I. General background and discovery of serotonin as a basis for 5-HT receptor identification. Pharmacol. Rep..

[39] Green A.R. (2006). Neuropharmacology of 5-hydroxytryptamine. Br. J. Pharmacol..

[40] Mohammad-Zadeh L.F., Moses L., Gwaltney-Brant S.M. (2008). Serotonin: A review. J. Vet. Pharmacol. Ther..

[41] Songtachalert T., Roomruangwong C., Carvalho A.F., Bourin M., Maes M. (2018). Anxiety Disorders: Sex Differences in Serotonin and Tryptophan Metabolism. Curr. Top. Med. Chem..

[42] Zhuang X., Xu H., Fang Z., Xu C., Xue C., Hong X. (2018). Platelet serotonin and serotonin transporter as peripheral surrogates in depression and anxiety patients. Eur. J. Pharmacol..

[43] Zmudzka E., Salaciak K., Sapa J., Pytka K. (2018). Serotonin receptors in depression and anxiety: Insights from animal studies. Life Sci..

[44] Mawe G.M., Hoffman J.M. (2013). Serotonin Signaling in the Gastrointestinal Tract-Functions, dysfunctions, and therapeutic targets. Nat. Rev. Gastroenterol. Hepatol..

[45] Latorre E., Layunta E., Grasa L., Castro M., Alcalde A.I., Mesonero J.E. (2016). Intestinal Serotonin Transporter Inhibition by Toll-Like Receptor 2 Activation. A Feedback Modulation. PLoS ONE.

[46] Thijssen A.Y., Mujagic Z., Jonkers D.M.A.E., Ludidi S., Keszthelyi D., Hesselink M.A., Clemens C.H., Conchillo J.M., Kruimel J.W., Masclee A.A. (2016). Alterations in serotonin metabolism in the irritable bowel syndrome. Aliment. Pharmacol. Ther..

[47] Gershon M.D., Tack J. (2007). The Serotonin Signaling System: From Basic Understanding to Drug Development for Functional GI Disorders. Gastroenterology.

[48] Otoshi C.K., Walwyn W.M., Tillakaratne N.J.K., Zhong H., Roy R.R., Edgerton V.R. (2009). Distribution and localization of 5-HT(1A) receptors in the rat lumbar spinal cord after transection and deafferentation. J. Neurotrauma.

[49] Breit S., Kupferberg A., Rogler G., Hasler G. (2018). Vagus Nerve as Modulator of the Brain-Gut Axis in Psychiatric and Inflammatory Disorders. Front. Psychiatry.

[50] Gunn D., Garsed K., Lam C., Singh G., Lingaya M., Wahl V., Niesler B., Henry A., Hall I.P., Whorwell P. (2019). Abnormalities of mucosal serotonin metabolism and 5-HT_3_ receptor subunit 3C polymorphism in irritable bowel syndrome with diarrhoea predict responsiveness to ondansetron. Aliment. Pharmacol. Ther..

[51] Chojnacki C., Błońska A., Kaczka A., Chojnacki J., Stępień A., Gąsiorowska A. (2018). Evaluation of serotonin and dopamine secretion and metabolism in patients with irritable bowel syndrome. Pol. Arch. Intern. Med..

[52] Adler J.R., Vahora I.S., Tsouklidis N., Kumar R., Soni R., Khan S. (2020). How Serotonin Level Fluctuation Affects the Effectiveness of Treatment in Irritable Bowel Syndrome. Cureus.

[53] Keszthelyi D., Troost F., Jonkers D.M., van Eijk H.M., Dekker J., Buurman W.A., Masclee A.A. (2015). Visceral hypersensitivity in irritable bowel syndrome: Evidence for involvement of serotonin metabolism--A preliminary study. Neurogastroenterol. Motil..

[54] Houghton L.A., Atkinson W., Whitaker R.P., Whorwell P.J., Rimmer M.J. (2003). Increased platelet depleted plasma 5-hydroxytryptamine concentration following meal ingestion in symptomatic female subjects with diarrhoea predominant irritable bowel syndrome. Gut.

[55] Yu F., Huang S., Zhang H., Ye H., Chi H.G., Zou Y., Lv R.X., Zheng X.B. (2016). Comparison of 5-hydroxytryptophan signaling pathway characteristics in diarrhea-predominant irritable bowel syndrome and ulcerative colitis. World J. Gastroenterol..

[56] Shi H.L., Liu C.H., Ding L.L., Zheng Y., Fei X.Y., Lu L., Zhou X.M., Yuan J.Y., Xie J.Q. (2015). Alterations in serotonin, transient receptor potential channels and protease-activated receptors in rats with irritable bowel syndrome attenuated by Shugan decoction. World J. Gastroenterol..

[57] Camilleri M., Atanasova E., Carlson P.J., Ahmad U., Kim H.J., Viramontes B.E., McKinzie S., Urrutia R. (2002). Serotonin-transporter polymorphism pharmacogenetics in diarrhea-predominant irritable bowel syndrome. Gastroenterology.

[58] Kerckhoffs A.P.M., Linde J.J.M., Akkermans L.M.A., Samsom M. (2012). SERT and TPH-1 mRNA expression are reduced in irritable bowel syndrome patients regardless of visceral sensitivity state in large intestine. Am. J. Physiol. Gastrointest. Liver Physiol..

[59] Jin D.C., Cao H.L., Xu M.Q., Wang S.N., Wang Y.M., Yan F., Wang B.M. (2016). Regulation of the serotonin transporter in the pathogenesis of irritable bowel syndrome. World J. Gastroenterol..

[60] Camilleri M. (2004). Is there a SERT-ain association with IBS?. Gut.

[61] Baj A., Moro E., Bistoletti M., Orlandi V., Crema F., Giaroni C. (2019). Glutamatergic Signaling along The Microbiota-Gut-Brain Axis. Int. J. Mol. Sci..

[62] Christmas D.M., Badawy A.A., Hince D., Davies S.J., Probert C., Creed T., Smithson J., Afzal M., Nutt D.J., Potokar J.P. (2010). Increased serum free tryptophan in patients with diarrhea-predominant irritable bowel syndrome. Nutr. Res..

[63] Heitkemper M.M., Han C.J., Jarrett M.E., Gu H., Djukovic D., Shulman R.J., Raftery D., Henderson W.A., Cain K.C. (2016). Serum Tryptophan Metabolite Levels During Sleep in Patients with and without Irritable Bowel Syndrome (IBS). Biol. Res. Nurs..

[64] De Ponti F. (2004). Pharmacology of Serotonin: What a Clinician Should Know. Gut.

[65] Pytliak M., Vargová V., Mechírová V., Felšöci M. (2011). Serotonin receptors—From molecular biology to clinical applications. Physiol. Res..

[66] Crowell M.D. (2001). The role of serotonin in the pathophysiology of irritable bowel syndrome. Am. J. Manag. Care.

[67] Terry N., Margolis K.G. (2017). Serotonergic Mechanisms Regulating the GI Tract—Experimental Evidence and Therapeutic Relevance. Handb. Exp. Pharmacol..

[68] Lacy B.E., Nicandro J.P., Chuang E., Earnest D.L. (2018). Alosetron use in clinical practice: Significant improvement in irritable bowel syndrome symptoms evaluated using the US Food and Drug Administration composite endpoint. Ther. Adv. Gastroenterol..

[69] El-Ayache N., Galligan J.J. (2019). 5-HT_3_ receptor signaling in serotonin transporter-knockout rats a female sex-specific animal model of visceral hypersensitivity. Am. J. Physiol. Gastrointest. Liver Physiol..

[70] Min Y.W., Rhee P.L. (2015). The clinical potential of ramosetron in the treatment of irritable bowel syndrome with. Ther. Adv. Gastroenterol..

[71] Eom S., Jung W., Lee J., Yeom H.D., Lee S., Kim C., Park H.D., Lee J.H. (2021). Differential Regulation of Human Serotonin Receptor Type 3A by Chanoclavine and Ergonovine. Molecules.

[72] Tack J., Coulie B., Wilmer A., Andrioli A., Janssens J. (2000). Influence of sumatriptan on gastric fundus tone and on the perception of gastric distension in man. Gut.

[73] Mulak A., Paradowski L. (2006). Effect of 5-HT1 agonist (sumatriptan) on anorectal function in irritable bowel syndrome patients. World J. Gastroenterol..

[74] James G.M., Baldinger-Melich P., Phillippe C., Kranz G.S., Vanicek T., Hahn A., Gryglewski G., Hienert M., Spies M., Traub-Weidinger T. (2017). Effects of Selective Serotonin Reuptake Inhibitors on Interregional Relation of Serotonin Transporter Availability in Major Depression. Front. Hum. Neurosci..

[75] Grover M., Camilleri M. (2013). Effects on gastrointestinal functions and symptoms of serotonergic psychoactive agents used in functional gastrointestinal diseases. J. Gastroenterol..

[76] Tack J., Broekaert D., Fischer B., Van Oudenhove L., Gevers A.M., Janssens J. (2006). A controlled crossover study of the selective serotonin reuptake inhibitor citalopram in irritable bowel syndrome. Gut.

[77] Ladabaum U., Sharabidze A., Levin T.R., Zhao W.K., Chung E., Bacchetti P., Jin C., Grimes B., Pepin C.J. (2010). Citalopram is not Effective Therapy for Non-Depressed Patients with Irritable Bowel Syndrome. Clin. Gastroenterol. Hepatol..

[78] Lin W., Liao Y., Peng Y.C., Chang C.H., Lin C.H., Yeh H.Z., Chang C.S. (2017). Relationship between use of selective serotonin reuptake inhibitors and irritable bowel syndrome: A population- based cohort study. World J. Gastroenterol..

[79] Kuiken S.D., Tytgat G.N.J., Boeckxstaens G.E.E. (2003). The selective serotonin reuptake inhibitor fluoxetine does not change rectal sensitivity and symptoms in patients with irritable bowel syndrome: A double blind, randomized, placebo-controlled study. Clin. Gastroenterol. Hepatol..

[80] Lacy B.E., Weiser K., De Lee R. (2009). The Treatment of Irritable Bowel Syndrome. Ther. Adv. Gastroenterol..

[81] Mujagic Z., Keszthelyi D., Thijssen A.Y., Jonkers D.M.A.E., Masclee A.A.M. (2016). Editorial: Serotonin and irritable bowel syndrome—Reconciling pharmacological effects with basic biology; authors’ reply. Aliment. Pharmacol. Ther..

[82] Zhou Y., Danbolt N.C. (2014). Glutamate as a neurotransmitter in the healthy brain. J. Neural. Transm..

[83] Fontana A.C.K. (2015). Current approaches to enhance glutamate transporter function and expression. J. Neurochem..

[84] Nakamura E., Uneyama H., Torii K. (2013). Gastrointestinal nutrient chemosensing and the gut-brain axis: Significance of glutamate signaling for normal digestion. J. Gastroenterol. Hepatol..

[85] Petroff O.A.C. (2002). GABA and glutamate in the human brain. Neuroscientist.

[86] Young V.R., Ajami A.M. (2000). Glutamate: An amino acid of particular distinction. J. Nutr..

[87] Peres M.F.P., Zukerman E., Soares C.A.S., Alonso E.O., Santos B.F.C., Faulhaber M.H.W. (2004). Cerebrospinal fluid glutamate levels in chronic migraine. Cephalalgia.

[88] Ramos-Vicente D., Ji J., Gratacòs-Batlle E., Reig-Viader R., Luís J., Burguera D., Navas-Perez E., García-Fernández J., Fuentes-Prior P., Escriva H. (2018). Metazoan evolution of glutamate receptors reveals unreported phylogenetic groups and divergent lineage-specific events. Elife.

[89] Goodwani S., Saternos H., Alasmari F., Sari Y. (2017). Metabotropic and ionotropic glutamate receptors as potential targets for the treatment of alcohol use disorder. Neurosci. Biobehav. Rev..

[90] Hornby P.J. (2001). Receptors and transmission in the brain-gut axis II. Excitatory amino acid receptors in the brain-gut axis. Am. J. Physiol. Gastrointest. Liver Physiol..

[91] Mathews D., Henter I., Zarate C.A. (2012). Targeting the Glutamatergic System to Treat Major Depressive Disorder. Drugs.

[92] Tsurugizawa T., Uematsu A., Nakamura E., Hasumura M., Hirota M., Kondoh T., Uneyama H., Torii K. (2009). Mechanisms of neural response to gastrointestinal nutritive stimuli. Gastroenterology.

[93] Chandra R., Liddle R.A. (2013). Modulation of pancreatic exocrine and endocrine secretion. Curr. Opin. Gastroenterol..

[94] Julio-Pieper M., Hyland N.P., Bravo J.A., Dinan T.G., Cryan J.F. (2010). A novel role for the metabotropic glutamate receptor-7: Modulation of faecal water content and colonic electrolyte transport in the mouse. Br. J. Pharmacol..

[95] Uneyama H. (2011). Nutritional and physiological significance of luminal glutamate-sensing in the gastrointestinal functions. Yakugaku Zasshi.

[96] Icenhour A., Tapper S., Bednarska O., Witt S.T., Tisell A., Lundberg P., Elsenbruch S., Walter S. (2019). Elucidating the putative link between prefrontal neurotransmission, functional connectivity, and affective symptoms in irritable bowel syndrome. Sci. Rep..

[97] Harris R.E., Sundgren P.C., Craig A.D., Kirshenbaum E., Sen A., Napadow V., Clauw D.J. (2009). Elevated insular glutamate in fibromyalgia is associated with experimental pain. Arthritis Reum..

[98] Bednarska O., Icenhour A., Tapper S., Witt S.T., Tisell A., Lundberg P., Elsenbruch S., Engström M., Walter S. (2019). Reduced excitatory neurotransmitter levels in anterior insulae are associated with abdominal pain in irritable bowel syndrome. Pain.

[99] Shao L., Liu Y., Ciao J., Wang Q., Liu F., Ding J. (2019). Activating metabotropic glutamate receptor-7 attenuates visceral hypersensitivity in neonatal maternally separated rats. Int. J. Mol. Med..

[100] Stachowicz K., Brañski P., Kłak K., van der Putten H., Cryan J.F., Flor P.J., Andrzej P. (2008). Selective activation of metabotropic G-protein-coupled glutamate 7 receptor elicits anxiolytic-like effects in mice by modulating GABAergic neurotransmission. Behav. Pharmacol..

[101] Filpa V., Moro E., Protasoni M., Crema F., Frigo G., Giaroni C. (2016). Role of glutamatergic neurotransmission in the enteric nervous system and brain-gut axis in health and disease. Neuropharmacology.

[102] Holton K.F., Taren D.L., Thomson C.A., Bennett R.M., Jones K.D. (2012). The effect of dietary glutamate on fibromyalgia and irritable bowel symptoms. Clin. Exp. Rheumatol..

[103] Zhou Q., Verne M.L., Fields J.Z., Lefante J.J., Basra S., Salameh H., Verne G.N. (2019). Randomised placebo-controlled trial of dietary glutamine supplements for postinfectious irritable bowel syndrome. Gut.

[104] Ferrigno A., Berardo C., Di Pasqua L.G., Siciliano V., Richelmi P., Vairetti M. (2017). Localization and role of metabotropic glutamate receptors subtype 5 in the gastrointestinal tract. World J. Gastroenterol..

[105] Moloney R.D., O’Mahony S.M., Dinan T.G., Cryan J. (2015). Stress-induced visceral pain: Toward animal models of irritable-bowel syndrome and associated comorbidities. Front. Psychiatry.

[106] Meymandi M.S., Keyhanfar F., Sepehri G.R., Heravi G., Yazdanpanah O. (2017). The Contribution of NMDA Receptors in Antinociceptive Effect of Pregabalin: Comparison of Two Models of Pain Assessment. Anesth. Pain Med..

[107] Bolton M.M., Pittman A.J., Lo D.C. (2000). Brain-derived neurotrophic factor differentially regulates excitatory and inhibitory synaptic transmission in hippocampal cultures. J. Neurosci..

[108] Roth F.C., Draguhn A. (2012). GABA metabolism and transport: Effects on synaptic efficacy. Neural Plast..

[109] Cuypers K., Maes C., Swinnen S.P. (2018). Aging and GABA. Aging.

[110] Siucinska E. (2019). Γ-Aminobutyric acid in adult brain: An update. Behav. Brain Res..

[111] Olsen R.W., Sieghart W. (2009). GABA A receptors: Subtypes provide diversity of function and pharmacology. Neuropharmacology.

[112] Tomita S. (2019). Molecular constituents and localization of the ionotropic GABA receptor complex in vivo. Curr. Opin. Neurobiol..

[113] Li Y., Xiang Y., Lu W., Liu C., Li J. (2012). A novel role of intestine epithelial GABAergic signaling in regulating intestinal fluid secretion. Am. J. Physiol. Gastrointest. Liver Physiol..

[114] Fischer A.U., Nicolas I.C.M., Deller T., Del Turco D., Fisch J.O., Griesemer D., Kattler K., Maraslioglu A., Roemer V., Xu-Friedman M.A. (2019). GABA is a modulator, rather than a classical transmitter, in the medial nucleus of the trapezoid body—Lateral superior olive sound localization circuit. J. Physiol..

[115] Naffaa M.M., Hung S., Chebib M., Johnston G.A.R., Hanrahan J.R. (2017). GABA-ρ receptors: Distinctive functions and molecular pharmacology. Br. J. Pharmacol..

[116] Zhou Y., Danbolt N.C. (2013). GABA and Glutamate Transporters in Brain. Front. Endocrinol..

[117] Loeza-Alcocer E., McPherson T.P., Gold M.S. (2019). Peripheral GABA receptors regulate colonic afferent excitability and visceral nociception. J. Physiol..

[118] Hyland N.P., Cryan J.F. (2010). A Gut Feeling about GABA: Focus on GABA B receptors. Front. Pharmacol..

[119] Aggarwal S., Ahuja V., Paul J. (2018). Dysregulation of GABAergic Signalling Contributes in the Pathogenesis of Diarrhea-predominant Irritable Bowel Syndrome. Neurogastroenterol. Motil..

[120] Harper D.E., Ichesco E., Schrepf A., Halvorson M., Puiu T., Clauw D.J., Harris R.E., Harte S.E., MAPP Research Network (2018). Relationships between brain metabolite levels, functional connectivity, and negative mood in urologic chronic pelvic pain syndrome patients compared to controls: A MAPP research network study. NeuroImage Clin..

[121] Zhang M.M., Liu S.B., Chen T., Koga K., Zhang T., Li Y.Q., Zhuo M. (2014). Effects of NB001 and gabapentin on irritable bowel syndrome-induced behavioral anxiety and spontaneous pain. Mol. Brain..

[122] Saito Y.A., Almazar A.E., Tilkes K.E., Choung R.S., Van Norstrand M.D., Schleck C.D., Zinsmeister A.R., Talley N.J. (2019). Randomised Clinical Trial: Pregabalin Versus Placebo for Irritable Bowel Syndrome. Aliment. Pharmacol. Ther..

[123] Needham K., Bron R., Hunne B., Nguyen T.V., Turner K., Nash M., Furnes S.J.B. (2010). Identification of subunits of voltage-gated calcium channels and actions of pregabalin on intrinsic primary afferent neurons in the guinea-pig ileum. Neurogastroenterol. Motil..

[124] Nissen T., Brock C., Lykkesfeldt J., Lindström E., Hultin L. (2018). Pharmacological modulation of colorectal distension evoked potentials in conscious rats. Neuropharmacology.

[125] Ma X., Sun Q., Sun X., Chen D., Wei C., Yu X., Liu C., Li Y., Li J. (2018). Activation of GABA_A_ Receptors in Colon Epithelium Exacerbates Acute Colitis. Front. Immunol..

[126] Park K.B., Ji G.E., Park M.S., Oh S.H. (2005). Expression of Rice Glutamate Decarboxylase in Bifidobacterium Longum Enhances γ-Aminobutyric Acid Production. Biotechnol. Lett..

[127] Minervini F., Bilancia M., Siragusa S., Gobbetti M., Caponio F. (2009). Fermented goats’ milk produced with selected multiple starters as a potentially functional food. Food Microbiol..

[128] Racke K., Matthiesen S. (2004). The airway cholinergic system: Physiology and pharmacology. Pulm. Pharmacol. Ther..

[129] Oda A., Tanaka H. (2014). Activities of nicotinic acetylcholine receptors modulate neurotransmission and synaptic architecture. Neural Regen. Res..

[130] Kruse A.C., Hu J., Pan A.C., Arlow D.H., Rosenbaum D.M., Rosemond E., Green H.F., Liu T., Chae P.S., Dror R.O. (2012). Structure and dynamics of the M3 muscarinic acetylcholine receptor. Nature.

[131] Brown D.A. (2019). Acetylcholine and cholinergic receptors. Brain Neurosci. Adv..

[132] Verma S., Kumar A., Tripathi T., Kumar A. (2018). Muscarinic and nicotinic acetylcholine receptor agonists: Current scenario in Alzheimer’s disease therapy. J. Pharm. Pharmacol..

[133] Picciotto M.R., Higley M.J., Mineur Y.S. (2012). Acetylcholine as a neuromodulator: Cholinergic signaling shapes nervous system function and behavior. Neuron.

[134] Yu A.J., Dayan P. (2005). Uncertainty, neuromodulation, and attention. Neuron.

[135] Cox M.A., Bassi C., Saunders M.E., Nechanitzky R., Morgado-Palacin I., Zheng C., Mak T.W. (2020). Beyond neurotransmission: Acetylcholine in immunity and inflammation. J. Intern. Med..

[136] Grossberg S. (2017). Acetylcholine Neuromodulation in Normal and Abnormal Learning and Memory: Vigilance Control in Waking, Sleep, Autism, Amnesia and Alzheimer’s Disease. Front. Neural Circuits.

[137] Deb B., Prichard D.O., Bharucha A.E. (2020). Constipation and Fecal Incontinence in the Elderly. Curr Gastroenterol. Rep..

[138] Russell J.P., Mohammadi E., Ligon C., Latorre R., Johnson A.C., Hoang B., Krull D., Ho M.W., Eidam H.S., DeMartino M.P. (2019). Enteric RET inhibition attenuates gastrointestinal secretion and motility via cholinergic signaling in rat colonic mucosal preparations. Neurogastroenterol. Motil..

[139] Hirota C.L., McKay D.M. (2006). Cholinergic regulation of epithelial ion transport in the mammalian intestine. Br. J. Pharmacol..

[140] Bonaz B., Bazin T., Pellissier S. (2018). The Vagus Nerve at the Interface of the Microbiota-Gut-Brain Axis. Front. Neurosci..

[141] Leng Y.X., Wei Y.Y., Chen H., Zhou S.P., Yang Y.L., Duan L.P. (2010). Alteration of cholinergic and peptidergic neurotransmitters in rat ileum induced by acute stress following transient intestinal infection is mast cell dependent. Chin. Med. J..

[142] Hod K., Sperber A.D., Maharshak N., Ron Y., Shapira I., David Z., Rogowski O., Berliner S., Shenhar-Tsarfaty S., Dekel R. (2018). Serum cholinesterase activity is elevated in female diarrhea-predominant irritable bowel syndrome patients compared to matched controls. Neurogastroenterol. Motil..

[143] Fujikawa Y., Tominaga K., Tanaka F., Tanigawa T., Watanabe T., Fujiwara Y., Arakawa T. (2015). Enteric glial cells are associated with stress-induced colonic hyper-contraction in maternally separated rats. Neurogastroenterol. Motil..

[144] Miampamba M., Million M., Yuana P.Q., Larauchea M., Tache Y. (2007). Water avoidance stress activates colonic myenteric neurons in female rats. Neuroreport.

[145] Peters S.A., Edogawa S., Sundt W.J., Dyer R.B., Dalenberg D.A., Mazzone A., Singh R.J., Moses N., Smyrk T.C., Weber C. (2017). Constipation-Predominant Irritable Bowel Syndrome Females Have Normal Colonic Barrier and Secretory Function. Am. J. Gastroenterol..

[146] Medland J.E., Pohl C.S., Edwards L.L., Frandsen S., Bagley K., Li Y., Moeser A.J. (2016). Early life adversity in piglets induces long-term upregulation of the enteric cholinergic nervous system and heightened, sex-specific secretomotor neuron responses. Neurogastroenterol. Motil..

[147] Balestra B., Vicini R., Cremon C., Zecchi L., Dothel G., Vasina V., De Giorgio R., Paccapelo A., Pastoris O., Stanghellini V. (2012). Colonic mucosal mediators from patients with irritable bowel syndrome excite enteric cholinergic motor neurons. Neurogastroenterol. Motil..

[148] Lin M.J., Yu B.P. (2018). Role of High-affinity Choline Transporter 1 in Colonic Hypermotility in a Rat Model of Irritable Bowel Syndrome. J. Neurogastroenterol. Motil..

[149] Zhao C., Lin M., Pan Y., Yu B. (2018). Blockage of High-Affinity Choline Transporter Increases Visceral Hypersensitivity in Rats with Chronic Stress. Gastroenterol. Res. Pract..

[150] Lin M.J., Yu B.P. (2018). Upregulation of the high-affinity choline transporter in colon relieves stress-induced hiperalgesia. J. Pain Res..

[151] Bharucha A.E., Ravi K., Zinsmeister A.R. (2010). Comparison of selective M3 and nonselective muscarinic receptor antagonists on gastrointestinal transit and bowel habits in humans. Am. J. Physiol. Gastrointest. Liver Physiol..

[152] Dumitrascu D.L., Chira A., Bataga S., Diculescu M., Drug V., Gheorghe C., Goldis A., Nedelcu L., Porr P.J., Sporea I. (2014). The Use of Mebeverine in Irritable Bowel Syndrome. A Position Paper of the Romanian Society of Neurogastroenterology based on Evidence. J. Gastrointestin. Liver Dis..

[153] Zheng L., Lai Y., Lu W., Li B., Fan H., Yan Z., Gong C., Wan X., Wu J., Huang D. (2015). Pinaverium Reduces Symptoms of Irritable Bowel Syndrome in a Multicenter, Randomized, Controlled Trial. Clin. Gastroenterol. Hepatol..

[154] De Schryver A.M.P., Samsom M. (2000). New developments in the treatment of Irritable Bowel Syndrome. Scand. J. Gastroenterol..

[155] Bharucha A.E., Seide B., Guan G., Andrews C., Zinsmeister A.R. (2008). Effect of tolterodine, on gastrointestinal transit and bowel habits in healthy subjects. Neurogastroenterol. Motil..

[156] Bulchandani S., Toozs-Hobson P., Parsons M., McCooty S., Perkins K., Latthe P. (2015). Effect of anticholinergics on the overactive bladder and bowel domain of the electronic personal assessment questionnaire (ePAQ). Int. Urogynecol. J..

[157] Houghton L.A., Rogers J.R., Whorwell P.J., Campbell F.C., Williams N.S., Goka J. (1997). Zamifenacin (UK-76, 654), a potent gut M 3 selective muscarinic antagonist, reduces colonic motor activity in patients with irritable bowel syndrome. Aliment. Pharmacol. Ther..

[158] Wong B.S., Camilleri M., Busciglio I., Carlson P., Szarka L.A., Burton D., Zinsmeister A.R. (2011). Pharmacogenetic Trial of a Cannabinoid Agonist Shows Reduced Bowel Syndrome. Gastroenterology.

[159] Mousavi T., Nikfar S., Abdollahi M. (2020). An update on efficacy and safety considerations for the latest drugs used to treat irritable bowel syndrome. Expert Opin. Drug Metab. Toxicol..

[160] Lieberman P. (2011). The basics of histamine biology. Ann. Allergy Asthma Immunol..

[161] Keshteli A.H., Madsen K.L., Mandal R., Boeckxstaens G.E., Bercik P., De Palma G., Reed D.E., Wishart D., Vanner S., Dieleman L.A. (2019). Comparison of the metabolomic profiles of irritable bowel syndrome patients with ulcerative colitis patients and healthy controls: New insights into pathophysiology and potential biomarkers. Aliment. Pharmacol. Ther..

[162] Hattori T., Watanabe S., Kano M., Kanazawa M., Fukudo S. (2010). Differential responding of autonomic function to histamine H 1 antagonism in irritable bowel syndrome. Neurogastroenterol. Motil..

[163] Deiteren A., De Man J.G., Ruyssers N.E., Moreels T.G., Pelckmans P.A., De Winter B.Y. (2014). Histamine H4 and H1 receptors contribute to postin fl ammatory visceral hypersensitivity. Gut.

[164] Wouters M.M., Balemans D., Van Wanrooy S., Dooley J., Cibert-Goton V., Alpizar Y.A., Valdez-Morales E.E., Nasser Y., Van Veldhoven P.P., Vanbrabant W. (2016). Histamine Receptor H1-Mediated Sensitization of TRPV1 Mediates Visceral Hypersensitivity and Symptoms in Patients with Irritable Bowel Syndrome. Gastroenterology.

[165] Klooker T.K., Braak B., Koopman K.E., Welting O., Wouters M.M., van der Heide S., Schemann M., Bischoff S.C., van den Wijngaard R.M., Boeckxstaens G.E. (2010). The mast cell stabiliser ketotifen decreases visceral hypersensitivity and improves intestinal symptoms in patients with irritable bowel syndrome. Gut.

[166] Tack J.F., Miner P.B., Fischer L., Harris M.S. (2011). Randomised clinical trial: The safety and efficacy of AST-120 in non-constipating irritable bowel syndrome—A double-blind, placebo-controlled study. Aliment. Pharmacol. Ther..

[167] Zizzo M.G., Bellanca A., Amato A., Serio R. (2020). Opposite effects of dopamine on the mechanical activity of circular and longitudinal muscle of human colon. Neurogastroenterol. Motil..

[168] Prakash S., Prakash A. (2021). Dopa responsive irritable bowel syndrome: Restless bowel syndrome or a gastrointestinal variant of restless legs syndrome?. BMJ Case Rep..

[169] Nozu T., Miyagishi S., Kumei S., Nozu R., Takakusaki K., Okumura T. (2019). Metformin inhibits visceral allodynia and increased gut permeability induced by stress in rats. J. Gastroenterol. Hepatol..

[170] Nozu T., Miyagishi S., Nozu R., Takakusaki K., Okumura T. (2019). Butyrate inhibits visceral allodynia and colonic hyperpermeability in rat models of irritable bowel syndrome. Sci. Rep..

[171] Latorre E., Mesonero J.E., Harries L.W. (2019). Alternative splicing in serotonergic system: Implications in neuropsychiatric disorders. J. Psychopharmacol..

[172] Carco C., Young W., Gearry R.B., Talley N.J., McNabb W.C., Roy N.C. (2020). Increasing Evidence That Irritable Bowel Syndrome and Functional Gastrointestinal Disorders Have a Microbial Pathogenesis. Front. Cell. Infect. Microbiol..

[173] Halkjær S.I., Christensen A.H., Lo B.Z.S., Browne P.D., Günther S., Hansen L.H., Petersen A.M. (2018). Faecal microbiota transplantation alters gut microbiota in patients with irritable bowel syndrome: Results from a randomised, double-blind placebo-controlled study. Gut.

